# Use of 5% Topical Minoxidil Application for Telogen Effluvium: An Open‐Label Single‐Arm Clinical Trial

**DOI:** 10.1111/1346-8138.17844

**Published:** 2025-07-02

**Authors:** Manabu Ohyama, Ryokichi Irisawa, Masaki Uchiyama, Miho Mori, Kazuhiro Aoki, Yuto Ueda, Toru Fujita

**Affiliations:** ^1^ Department of Dermatology Kyorin University School of Medicine Tokyo Japan; ^2^ Department of Dermatology Tokyo Medical University Tokyo Japan; ^3^ Clinical Development Self‐Medication, Taisho Pharmaceutical co., Ltd. Tokyo Japan

**Keywords:** COVID‐19, hair shedding, psychological stress, telogen effluvium, topical minoxidil

## Abstract

Telogen effluvium (TE) is an acute and diffuse hair loss due to an abnormal increase in telogen hair follicles in response to external or internal factors. Recently, TE attracts global interest as a major cause of hair loss as a sequela of COVID‐19. Typically, hair shedding in TE is expected to cease around 3–6 months from the onset with spontaneous hair regrowth, once the triggering factor is eliminated. However, the substantial shedding causes heavy psychological stress on patients, and there is no evidence‐based treatment. To assess the usefulness of topical minoxidil, which has been reported to improve the hair cycle, for TE, an open‐label, single‐arm clinical trial was conducted enrolling 12 Japanese subjects (3 men and 9 women) who were diagnosed as TE by experts. For each subject, 1 mL of 5% topical minoxidil lotion was applied to the entire scalp twice daily for 24 weeks. Phototrichogram detected that the terminal hair (≥ 60 μm in diameter) count markedly increased respectively by 12.55 ± 4.99 hairs/cm^2^ at week 4 and 11.20 ± 4.79 hairs/cm^2^ at week 12 from baseline. In the hair wash test assessment, nearly 70% of the subjects were evaluated as improved at least by 2 grades in the evaluation scale (more than 100 shed hair decrease in the count) from baseline. Of note, all investigators and subjects reported notable improvement at week 24 respectively in their reported outcomes. Adverse event was minimal and within the known safety profile of topical minoxidil. Self‐healing nature of TE and small sample size represented major factors affecting the validity of the study. Considering its safety profile and wide‐availability, topical minoxidil lotion may be helpful in the management of TE making the patients reassured at earlier stage of clinical course. Still, it should be noted that this application is an off‐label use.

## 1 Introduction

Telogen effluvium (TE) is characterized by an increase in telogen hair follicles, leading to excessive club hair shedding due to external or internal insults such as febrile illness, childbirth, or psychological stress [1]. Recently, TE attracts global interest as a major cause of hair loss as a sequela of after severe SARS‐CoV‐2 infection (COVID‐19) possibly as a consequence of cytokine storms [2]. Typically, hair shedding in the acute (common) form of TE is expected to cease around 3–6 months from the onset, once the triggering factor is eliminated [3]. However, substantial hair shedding can lead to significant psychological stress and markedly reduce the patients' QOL [4].

Nevertheless, no evidence‐based treatment is currently available for reducing the disease burden of TE [5]. Despite felt want of the affected individuals for efficacious modalities, reassurance of hair recovery and counseling have been regarded as the first‐line treatment for TE [3]. Thus, any possible intervention can be beneficial for reducing the anxiety of TE patients.

In this study, we attempted to evaluate the usefulness of topical minoxidil, which is widely used for treating male and female pattern hair loss, for the management of TE patients. Topical minoxidil has been shown to promote hair growth via anagen induction presumably due to the activation of sulfonylurea receptors with resultant opening of vascular smooth muscle ATP‐sensitive K+ channels to improve hair follicle blood flow and the production of cell growth factors such as vascular endothelial growth factor from dermal papilla cells [6], and/or the inhibition of dermal papilla cell apoptosis [7]. Based on these previously suggested mode‐of‐action, topical minoxidil may accelerate the transition from telogen to anagen phase and thereby contribute to faster improvement of TE. In addition, the safety profile of topical minoxidil is well‐established, further highlighting an advantage of this modality as a supportive therapy for TE.

Based on these considerations, we conducted a 24‐week open‐label, single‐arm clinical trial to assess the usefulness of 5% topical minoxidil application for the subjects with excessive hair shedding and clinically diagnosed as TE secondary to various triggering events, including COVID‐19.

## 2 Methods

### 2.1 Subjects

Adult Japanese men and women with the claim of excessive shedding after having experienced known TE triggers (febrile illnesses including COVID‐19, crash diet, psychological stress, and other factors) [1, 2, 3] were subjected to physical examination by board‐certified dermatologists with more than 7‐year experience of managing hair diseases at specialized clinics. In this study, the diagnosis of TE was made based on the following criteria: (1) TE triggers must be identified, (2) the presence of excessive hair loss comprehensively indicates TE as judged by the investigator, (3) the absence of other types of hair loss and (4) no use of drugs potentially causing TE [8], and those satisfied these criteria were enrolled. The subjects underwent laboratory examination [Thyroid stimulating hormone, thyroid hormone (FT3, FT4), sex hormone (testosterone), prolactin, adrenocorticotropic hormone, cortisol, HBs antigen, HCV antibody, HIV antigen/antibody, syphilis qualitative (TP antibody method/RPR method), and antinuclear antibody] to rule out the presence of underlying conditions potentially causing hair loss, of note, subclinical endocrinological or immunological and infectious diseases [8]. The study protocol and informed consent form were reviewed and approved by the Review Board of Human Rights and Ethics for Clinical Studies Institutional Review Board (Tokyo, Japan). Each subject was given a sufficient explanation before participating in the study and signed the consent form prior to the enrollment.

### 2.2 Study Design

This study was conducted as a single center, open‐label, and single‐arm trial to assess the usefulness of 5% topical minoxidil application for TE. The investigational product was a 5% minoxidil topical lotion (containing 50 mg of minoxidil per 1 mL, along with secondary ingredients such as pyridoxine hydrochloride, tocopherol acetate, l‐menthol, hinokitiol, glycyrrhetinic acid, and diphenhydramine hydrochloride), 1 mL of which was applied to the entire scalp twice daily for 24 weeks. The subjects visited the clinic every 4 weeks (and at study discontinuation) to undergo efficacy assessments, including hair count and telogen hair ratio per 1 cm^2^ measured by phototrichogram. Evaluations included the amount of hair loss evaluated by a hair wash test and improvement assessments by both the dermatologists and the subjects compared to the baseline at the start of topical minoxidil treatment. At each visit, the subjects underwent examinations for adverse events, blood pressure and pulse rate measurements, laboratory testing (general blood tests, blood biochemistry, and urinalysis), and 12‐lead electrocardiograms.

During the study, the subjects were prohibited from using other hair growth products and topical treatments on the scalp, anti‐androgen medications, nonbreathable wigs, hair transplants, hair extensions, or specialized hair care (e.g., hair growth treatment massages). A subject was withdrawn from the study if the investigator deemed continued participation infeasible due to adverse events or other factors.

This study was conducted at Clinical Research Hospital Tokyo (Tokyo, Japan) from February 2022 to November 2023.

### 2.3 Phototrichogram

Hairs within a rectangular area (approximately 2 cm^2^) located 4–5 cm to the left or right of each subject's scalp midline were shaved to a length of about 0.8–1.0 mm. During each visit every 4 weeks, the same area was identified and shaved similarly to secure stereotaxis. This study used H2H (Hair‐to‐Hair) matching technology and virtual tattoo (TrichoLAB GmbH, Bad Birnbach, Germany) to identify the same evaluation area without using tattoos. The evaluation area was imaged using FotoFinder leviacam (FotoFinder GmbH, Bad Birnbach, Germany). The captured images were submitted to TrichoLAB GmbH (Bad Birnbach, Germany) for centralized measurement of hair counts and telogen hair ratio.

### 2.4 Hair Count

The number of hairs per 1 cm^2^ in the shaved area was measured by phototrichogram every 4 weeks from baseline to week 24. Hairs were categorized by thickness into vellus hairs (< 40 μm in diameter), nonvellus hairs (≥ 40 μm in diameter), terminal hairs (≥ 60 μm in diameter), and nonterminal hairs (< 60 μm in diameter) and counted for respective subgroups.

### 2.5 Telogen Hair Ratio Measurement

The telogen hair ratio per 1 cm^2^ in the shaved area was measured by phototrichogram every 4 weeks from baseline to week 24. To assess the elongation of each hair, a tested area was imaged twice at 48‐ to 72‐h intervals on each visit. Hairs with a growth rate of 0.1 mm/day or more were classified as anagen hairs, while those with less than 0.1 mm/day growth were classified as telogen hairs, allowing the calculation of the telogen hair ratio.

### 2.6 Hair Wash Test

Every 4 weeks from the baseline to week 24, the subjects were requested to collect hairs shed during shampooing at home over two consecutive days immediately prior to each visit, ensuring that the hairs from each day were respectively collected and stored separately. Subjects and dermatologists independently assessed the amount of collected hairs for each day by comparing them with hair samples (Figure S1) using a 10‐point scale (Table 1). Six types of hair samples for different hair lengths were prepared for accurate measurement. Both subjects and dermatologists evaluated the amount of shed hairs independently using the same hair length scale for consistency. This method is based on the approach originally invented by Sinclair R [9], which has been reported to be reliable with a high correlation coefficient between independent assessments of the same samples.

**TABLE 1:** Evaluation criteria of hair wash test.

Grade	Number of hairs collected
I	< 10
II	10 ≤ and < 50
III	50 ≤ and < 100
IV	100 ≤ and < 150
V	150 ≤ and < 200
VI	200 ≤ and < 250
VII	250 ≤ and < 300
VIII	300 ≤ and < 400
IX	400 ≤ and < 500
X	500 ≤

### 2.7 Hair Change Assessment and the Investigator Reported Outcome

During the study, the investigators (dermatologists) examined the scalp condition every 4 weeks up to week 24 (or at the time of discontinuation). Photographs of the entire scalp (global photo images) were taken at every visit. The change in hairs was assessed by comparing global photo images (global photographic assessment), hair wash test grades (investigators' assessment), and clinical findings at baseline and on each visit. The global investigator‐reported outcome was made using a 5‐point scale based on these changes in hairs: greatly improved, moderately improved, slightly improved, no change, or worsened.

### 2.8 Patient Reported Outcome

Every 4 weeks up to week 24 (or at the time of discontinuation), the subjects were asked to evaluate the changes in hair volume and the amount of hair shedding compared to baseline based on their own impression, without reference to the global photo images or the results of the hair wash tests. The change in hair volume was rated based on a five‐point scale: greatly increased, moderately increased, slightly increased, no change, or decreased. The change in the amount of hair shedding was rated using a five‐point scale: greatly decreased, moderately decreased, slightly decreased, no change, or increased.

### 2.9 Safety Evaluation

Prior to the enrollment, the investigators confirmed each subject's health status, medical history, comorbidities, and any hypersensitivity to medications. On each visit, a medical interview, physical examination particularly focusing on the scalp condition, and laboratory examination (general blood tests, blood biochemistry, and urinalysis), blood pressure and pulse rate measurements, and 12‐lead electrocardiograms were conducted.

### 2.10 Statistical Analysis

The one‐sample *t*‐test was conducted using SAS (version 9.3, SAS Institute, Cary, NC, USA). Estimated values and bilateral 95% confidence intervals were calculated with a significance level of 5% (two‐sided). Graphs and correlation coefficients were generated using Microsoft Excel (version 2202, Microsoft, Redmond, WA, USA).

## 3 Results

### 3.1 Subjects

Among the total of 95 candidates with the claims of unusual hair shedding screened, 12 subjects were found to be eligible for the enrollment and administered topical 5% minoxidil lotion for the efficacy and safety evaluation (Table 2). The cohort consisted of 3 males and 9 females with the average age of 47.7 ± 8.9 (33–63, mean ± SD). Five out of 12 subjects experienced COVID‐19. Other triggering events were psychological stress (*n* = 4), febrile illness (excluding COVID‐19) (*n* = 1), crash diet (*n* = 1), and appendectomy (*n* = 1). The average duration between the onset of excessive hair shedding and the initial visit was 15.5 ± 7.4 (7–30, mean ± SD) weeks, excluding one (TR‐01) who suffered from COVID‐19 16 weeks before the initial visit with ambiguously started hair shedding afterward. Two subjects discontinued the study: a 56‐year‐old female discontinued before week 12 due to contact dermatitis (TR‐04) and a 45‐year‐old female requested discontinuation of her own motive at week 20 (TR‐12).

**TABLE 2:** Subject's background.

Subject ID	Sex	Age	Factors causing telogen effluvium	Onset period (The time when hair loss symptoms began) [The number of weeks counted backward]	Hair count					Telogen hair ratio [%]	Hair wash test	
					Non vellus [/cm^2^]	Vellus [/cm^2^]	Terminal [/cm^2^]	Nonterminal [/cm^2^]	Total [/cm^2^]		Investigator	Subject
TR‐01	Male	48	Febrile illness (COVID‐19)	Unclear (visited 16 weeks after COVID‐19)	107.8	54.1	79.7	82.2	161.9	18.1	VI	VI
TR‐02	Male	50	Appendectomy	16	167.3	28.1	142.7	52.7	195.4	12.1	IV	VI
TR‐03	Female	45	Febrile illness (COVID‐19)	10	164.9	69.9	133.9	100.9	234.7	12.2	VI	VI
TR‐04	Female	56	Psychological stress	30	128.0	50.2	54.6	123.5	178.1	5.4	IV	IV
TR‐05	Male	36	Psychological stress	25	165.4	39.9	149.6	55.6	205.2	4.6	II	II
TR‐06	Female	63	Crash diet	17	127.0	32.5	96.5	63.0	159.4	7.2	VII	V
TR‐07	Female	42	Febrile illness (COVID‐19)	20	195.9	37.9	135.8	97.9	233.8	10.0	III	II
TR‐08	Female	55	Psychological stress	12	191.4	48.7	126.5	113.7	240.2	16.3	II	IV
TR‐09	Female	57	Febrile illness (excluding COVID‐19)	7	194.9	9.8	169.3	35.4	204.7	11.9	VIII	IV
TR‐10	Female	42	Febrile illness (COVID‐19)	17	220.0	19.2	190.0	49.2	239.2	5.9	V	III
TR‐11	Female	33	Psychological stress	8	108.8	50.2	86.1	72.8	159.0	27.8	I	I
TR‐12	Female	45	Febrile illness (COVID‐19)	8	217.0	66.9	133.9	150.1	284.0	17.1	II	II

*Note:* Subject demographics and hair parameters (hair counts, telogen hair ratio and hair wash test) at the time of study enrollment are shown. The onset time (the time when hair loss symptoms began) refers to the period before the baseline. As for the hair wash test, since TR‐11 only washed her hair lightly with warm water, so the grade was I.

### 3.2 Changes in Hair Count

**FIGURE 1:**
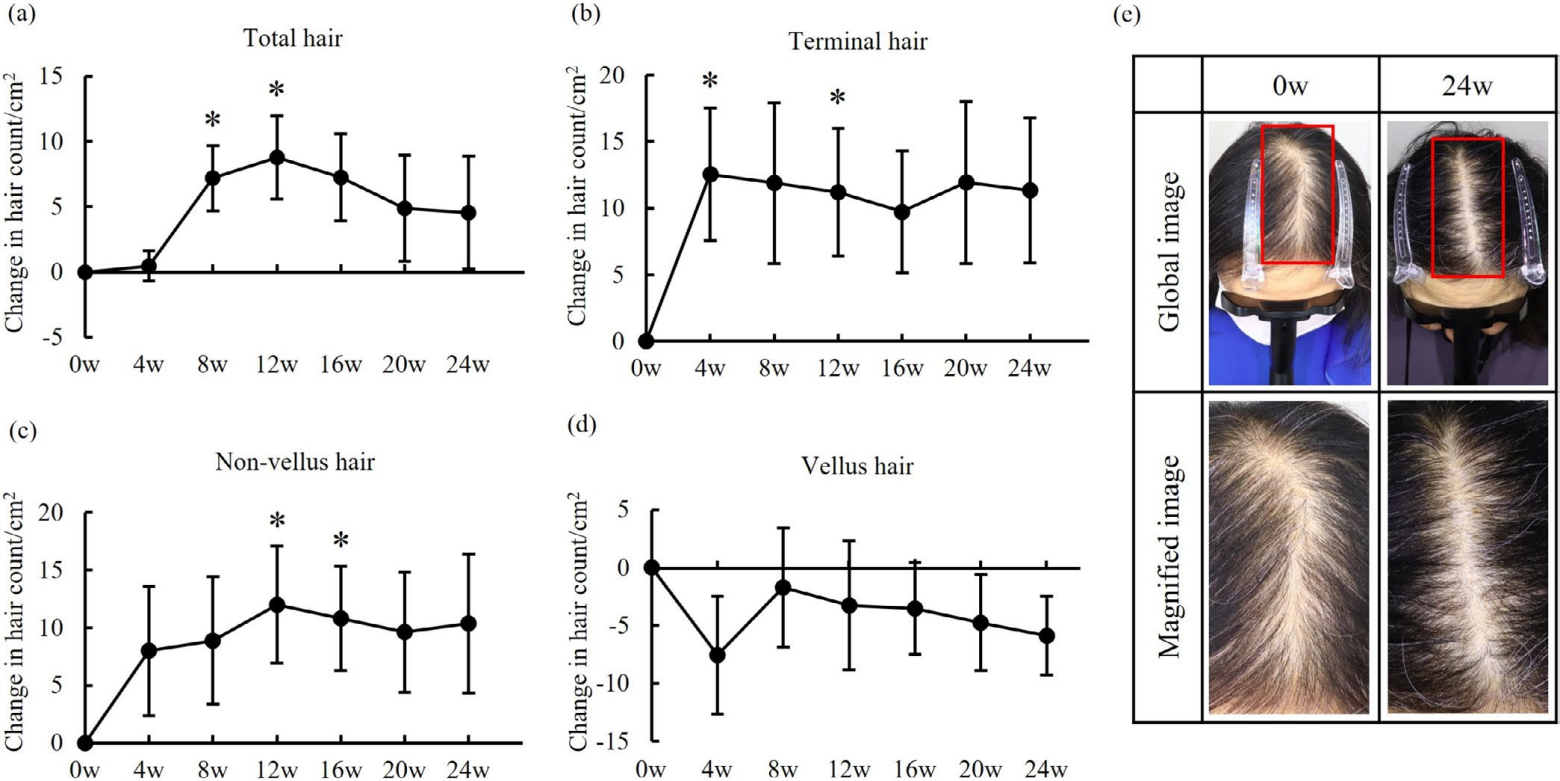
Changes in hair count. Changes in each hair count ((a) Total hair, (b) Terminal hair, (c) Nonvellus hair, (d) Vellus hair) after 5% minoxidil topical lotion administration in telogen effluvium. Bars and whiskers indicate means and standard errors (*p < 0.05; Paired *t*‐test). 0w (*n* = 12), 4w (*n* = 12), 8w (*n* = 11), 12w (*n* = 11), 16w (*n* = 11), 20w (*n* = 10), 24w (*n* = 10). w, week. (e) Global images of a subject who showed significant improvement in hair counts (TR‐06). The red boxes indicate the magnified areas.

Changes in hair count from baseline to week 24 are shown in Figure 1 (those of individual subjects were shown in Table S1). Total hair count showed a significant increase of 7.18 ± 2.49 (mean ± SE) hairs/cm^2^ and 8.75 hairs/cm^2^ respectively at week 8 and 12, compared to baseline (*p* < 0.05; Figure 1a). Of note, change in terminal hair count markedly increased by 12.55 ± 4.99 hairs/cm^2^ at week 4 and 11.20 ± 4.79 hairs/cm^2^ at week 12 from baseline (*p* < 0.05; Figure 1b). In addition, nonvellus hair count significantly increased by 12.01 ± 5.07 hairs/cm^2^ and 10.82 ± 4.51 hairs/cm^2^ at week 12 and 16 from baseline (*p* < 0.05; Figure 1c) with inverse decrease in vellus hair count (Figure 1d). A representative responder's global photo image was presented in Figure 1e. In this subject, total hair count increased by 24.3 hairs/cm^2^ at week 24 compared to baseline (Table S1). Individual changes in telogen hair ratios for each subject were demonstrated in Figure S2. Despite that all subjects were clinically diagnosed as TE by the dermatologists who expertized in the management of hair diseases, a wide variation in baseline telogen hair ratios was observed and no consistent trend in the changes of telogen hair ratio was readily detectable by FotoFinder leviacam system across the subjects.

### 3.3 Hair Wash Test

**FIGURE 2:**
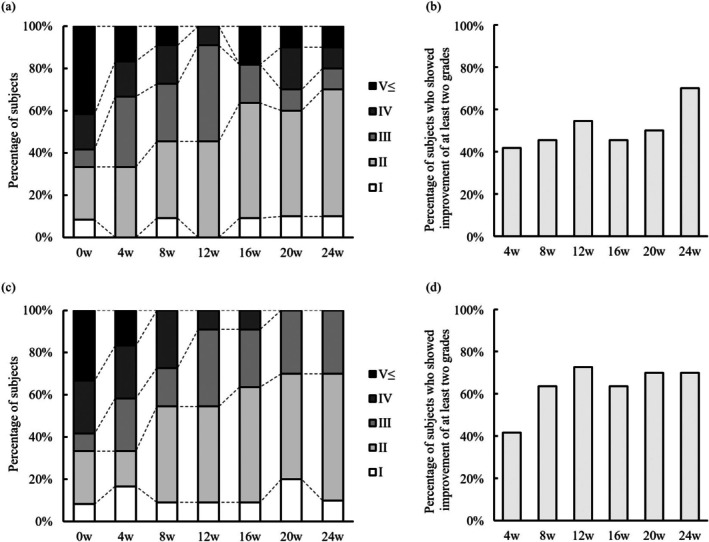
Results of the hair wash test. The results of the hair wash test, assessed using a 10‐point scale presented in Table 1, were shown. (a) Evaluation of the hair wash test by the dermatologists. Bars represent the percentage relative to the total number of evaluations. (b) Bars show the percentage of the subjects who achieved two or more grade improvement as assessed by the investigators. (c) Evaluation of the hair wash test by the subjects. Bars represent the percentage relative to the total number of evaluations. (d) Bars show the percentage of the subjects who achieved two or more grade improvement as assessed by the subjects. 0w (*n* = 12), 4w (*n* = 12), 8w (*n* = 11), 12w (*n* = 11), 16w (*n* = 11), 20w (*n* = 10), 24w (*n* = 10). w, week.

The samples collected by the hair wash test were evaluated by both the dermatologists and the subjects on each visit during the study period from baseline to Week 24 (Figure 2a–d). In the dermatologists' assessment, nearly 60% of the subjects had grades greater than IV at baseline. Nearly 40% of the subjects with grade greater than V at baseline, which consistently decreased approximately by half during baseline‐Week 4 ‐and Week 4‐8 (Figure 2a). More than 40% of the subjects achieved the improvement by the grade greater than 2 compared to the grade on previous visits with a tendency for progressive improvement over the study period (Figure 2b). The outcome was almost analogous in the subjects' assessment, supporting the reproducibility of the grading scale (Figure 2c,d). Intriguingly, the improvement as assessed by the subjects was consistently greater than that evaluated by the dermatologists, as demonstrated by the earlier disappearance of those with grades greater than V and the increase in those who achieved improvement of at least 2 grades from baseline (Figure 2c,d). The percentage of the subjects with improvement of at least 2 grades from baseline reached 70.0% in both in the dermatologists' and subjects' assessment (Figure 2b,d).

### 3.4 Investigator and Patient Reported Outcome

**FIGURE 3:**
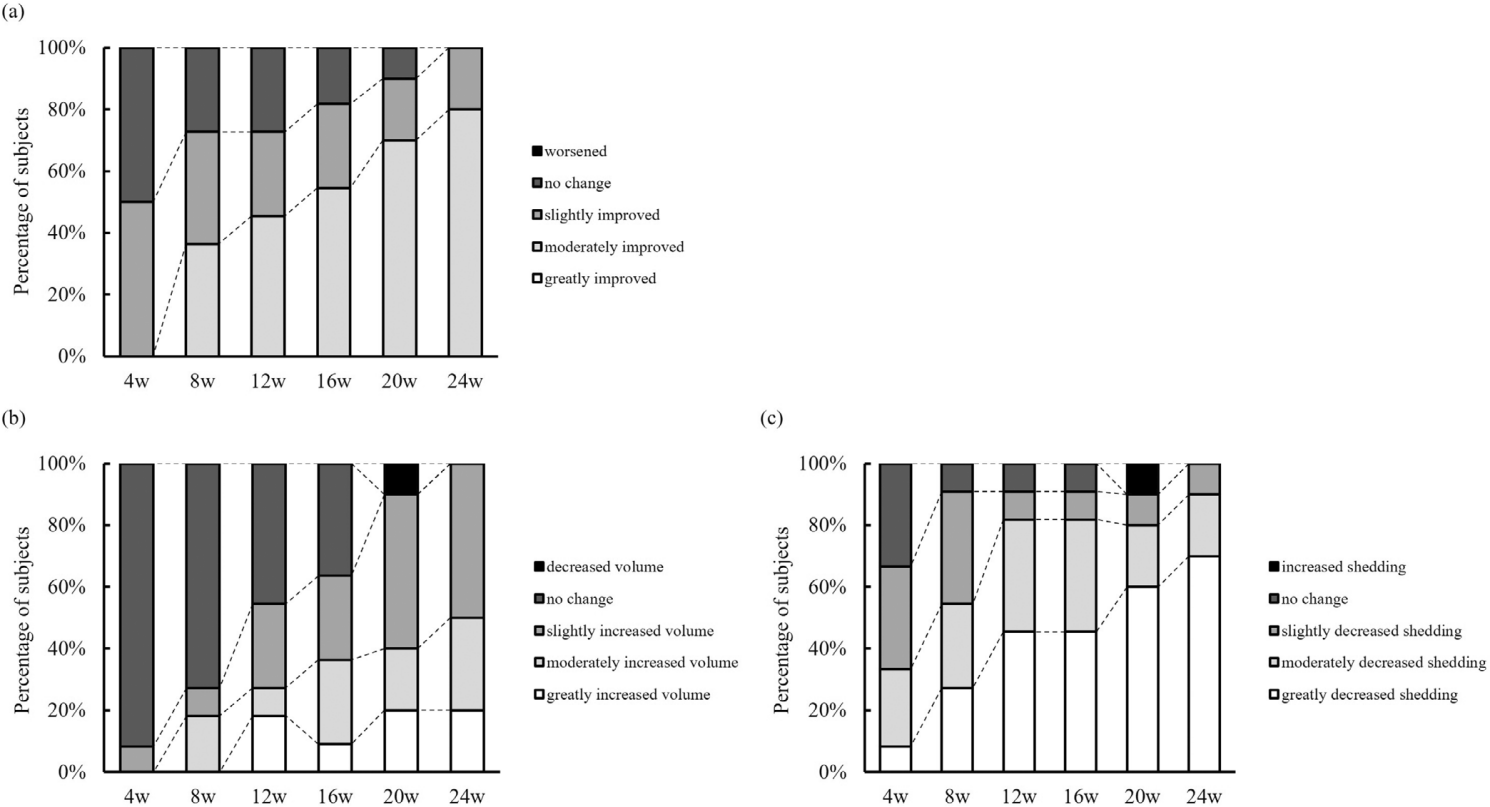
Results of investigator and patient reported outcome. The results of the investigator and patient (subject) reported outcome after 5% minoxidil topical lotion administration for telogen effluvium were shown. (a) The investigators' reported outcome of the improvement assessment. Bars represent the percentage relative to the total number of evaluations. (b, c) The patient reported outcome of hair volume (b) and shedding (c) assessment. Bars represent the percentage relative to the total number of evaluations. 4w (*n* = 12), 8w (*n* = 11), 12w (*n* = 11), 16w (*n* = 11), 20w (*n* = 10), 24w (*n* = 10). w, week.

The results for investigator and patient reported outcomes are presented in Figure 3. As the period of 5% minoxidil administration became longer, both investigator and patient reported outcomes tended to improve (Figure 3a–c). At Week 24, the proportion of subjects rated as “moderately improved” or better by investigators was 80.0%, and that of “slightly improved” or better was 100.0% (Figure 3a). Additionally, at Week 24, 50.0% of subjects perceived “moderately increased” or greater, and 100.0% perceived “slightly increased” or greater in their hair volume (Figure 3b). Similarly, with regards to shedding, 90.0% of subjects perceived “moderately decreased” or greater, and 100.0% perceived at least “slightly decreased” at Week 24 in their hair shedding (Figure 3c). These results indicate high satisfaction levels among both investigators and subjects for 5% minoxidil administration in TE. Notably, by Week 8, 72.7% of subjects were rated as “slightly improved” or better by investigators (Figure 3a), and 90.9% of subjects reported “slightly decreased” or better in their hair shedding (Figure 3c), suggesting relatively early observable improvements with 5% minoxidil use in TE.

### 3.5 Safety Evaluation

In this study, a total of 8 adverse events (in 5 subjects) were observed throughout the study period with a single adverse event (1 subject) judged to be directly related to the use of topical minoxidil application (Table 3). The reported adverse events included pyrexia, abdominal pain, vomiting, fibula fracture, contact dermatitis, increase in serum aspartate aminotransferase, or C‐reactive protein level, and glucose urine present; all of which were recovered. Fibula fracture was regarded as a major adverse event which was determined not to be directly related to the investigational product. In a subject with contact dermatitis, which is a known adverse event of topical minoxidil, the study was discontinued based on the dermatologists' judgment. Accordingly, topical minoxidil was well tolerated by the subjects clinically diagnosed as TE.

**TABLE 3:** Adverse events.

Subject ID	Sex	Age	Adverse event	Severity	Seriousness	Causality	Treatment of Investigational drug	Outcome
TR‐03	Female	45	Fibula fracture	Severe	Serious	Not related	No change	Resolved
TR‐04	Female	56	Contact dermatitis	Moderate	Nonserious	Related	Termination	Resolved
TR‐05	Male	36	Increase in serum aspartate aminotransferase	Mild	Nonserious	Not related	No change	Resolved
			Increase in serum C‐reactive protein level	Mild	Nonserious	Not related	No change	Resolved
			Pyrexia	Mild	Nonserious	Not related	No change	Resolved
TR‐11	Female	33	Glucose urine present	Mild	Nonserious	Not related	No change	Resolved
TR‐12	Female	45	Vomiting	Mild	Nonserious	Not related	No change	Resolved
			Abdominal pain	Mild	Nonserious	Not related	No change	Resolved

*Note:* All adverse events observed during the study period. The age is as of the time of consent acquisition. Severity was medically evaluated in three grades: mild, moderate, and severe. Seriousness was assessed in two categories: serious and nonserious.

## 4 Discussion

Recently, remarkable progress has been made in our understanding of the pathophysiology of hair diseases, leading to the invention of new therapeutic approaches represented by Janus kinase inhibitors for severe alopecia areata [10]. TE has been recognized as an established entity since the first description by Kligman [1]; however, most likely because of its self‐healing nature, the condition attracts little interest in clinical dermatology. TE has gained attention as a major cause of hair loss after COVID‐19 [2] making clinicians reminded of this condition. The prognosis of TE is usually favorable; hair loss will recover once triggering events are eliminated [3, 5]. Still, the fear of sudden hair shedding which may last for several months until the initiation of new anagen phase can cause the significant psychological distress to the affected individuals [4]. Therefore, improving TE at an early stage is crucial to improve QOL of patients and clear demand exists for therapeutic modality of this condition.

TE has been recognized as an established entity. Despite the reports of tests or examinations possibly serving as diagnostic tools for TE [11], our literature review found no definitive diagnostic criteria exists for this condition. TE has been reported to be heterogeneous with five distinct types of pathogenesis proposed; immediate anagen release, delayed anagen release, short anagen, immediate telogen release, and delayed telogen release [12]. This can make the diagnosis challenging [5]. Indeed, only 12.6% of candidates were identified as eligible for this study. In addition, telogen hair ratio was variable among the subjects and 4 of 12 subjects had a telogen hair ratio below the normal threshold of 10% [13, 14] at baseline. This could be explained by the variance in individual baselines under normal condition among the subjects and the differences in the pathomechanism underlying TE; evident increase in telogen hair ratio is detectable by trichoscopy‐based phototrichogram (time‐relapse imaging) in immediate anagen release, but not in delayed anagen release or immediate telogen release. The variable time‐course change in telogen hair ratio could also be attributed to these differences intrinsically accompanying TE. Still, the observation that the decrease in hair shedding was observed around 2–4 months after the recruitment suggested that typical TE patients were appropriately selected in this study.

Considering the complexity underlying TE, a therapeutic modality increasing anagen hair follicles or accelerating anagen entry would be of potential value. With its pharmacological effects on the hair cycle to promote the anagen phase, minoxidil can be a preferable treatment option for TE. Indeed, several studies have explored the use of oral minoxidil for the treatment of TE, with the outcomes supporting its effectiveness [15, 16]. However, oral minoxidil is not officially approved in Japan. In addition, based on the latest version of the Japanese Dermatological Association's management guideline for male/female pattern hair loss, oral minoxidil is not recommended as a treatment for hair loss [17], considering its potential adverse events such as pericardial effusion [18]. In contrast, topical minoxidil lotion is used worldwide for treating male and female pattern hair loss, and its safety profile is well‐established, providing a rationale for conducting this study. At the same time, it should be noted that topical minoxidil is known to cause paradoxical initial hair shedding, as suggested by the temporal increase in the telogen hair ratio or decrease in the number of terminal hairs observed in some patients in this study. This may lead to discontinuation of topical minoxidil and therefore patients need to be informed of this possibility prior to use [19] even in the context of TE.

Because of self‐healing nature of TE and variable duration from the onset, the assessment of *bona fide* efficacy of topical minoxidil application is technically challenging even with the use of strict and quantitative assessment methodologies including trichoscopy‐based phototrichogram. Still, the increase in total and terminal hair count became detectable 4 weeks after the initiation of the study. These changes were accompanied by the improvement observed in hair wash test. This test can be impressionistic by nature; a design which may lead to the inconsistency of the outcomes. Still, in the original report of the methodology adopted in this study, the technique found to be fairly reproducible, with a high correlation coefficient of 0.98 between independent assessments achieved even by patients [9]. Therefore, the results obtained in this study would be of some relevance. Considering that termination of hair shedding and hair regrowth can gradually take place within 3–6 months in TE, especially in acute TE [1, 3], it would be not overstating to conclude that topical minoxidil tendentiously accelerated anagen reentry and promoted the recovery from TE. As psychological stress due to excessive hair shedding represent a major problem of TE [4], the evaluation of patient reported outcomes is particularly important in TE management. It should be noted that, in both hair volume and shedding self‐evaluation by the subjects, the assessment outcome of “greatly improved” was respectively found at week 12 and 4, while none of the investigators selected this category in their evaluation. The aforementioned outcomes may partially reflect the subjects' relief or confidence coming from the use of topical minoxidil lotion, in addition to its real pharmacological effects.

We are aware of the major limitations of the study: a small sample size, an open‐label single‐arm design, and the heterogeneity and self‐healing nature of TE status. Unlike other hair diseases, TE is usually a transient condition with a variety of triggers and pathogenesis, and therefore it is technically challenging to collect a sufficient number of subjects with the same etiology and phase. As mentioned above, the majority of the volunteers screened were found to be ineligible for enrollment as they did not satisfy the criteria, further supporting the difficulty of conducting TE clinical studies. In addition, the affected individuals are usually anxious for treatments, making it difficult to conduct a placebo‐controlled study. Strictly adjusting a phase of TE could be possible by assessing the telogen hair ratio and the amount of hair shedding; however, this could make enrollment extremely difficult and impractical.

Taken together, considering its safety profile and wide‐availability, topical minoxidil lotion may be helpful in the management of TE making the patients reassured at earlier stage of clinical course. Still, it should be clearly noted that this is currently an off‐label use and further accumulation of the data by clinical trials with high‐level evidence is indispensable to draw a definitive conclusion and fully justify its usage for TE.

## Ethics Statement

Approval of the research protocol by an Institutional Reviewer Board: This study was conducted in accordance with the Declaration of Helsinki and J‐GCP. The study protocol and informed consent form were reviewed and approved by the Review Board of Human Rights and Ethics for Clinical Studies Institutional Review Board (Tokyo, Japan).

## Consent

Each subject was given a sufficient explanation before participating in the study and signed the consent form prior to the enrollment.

## Conflicts of Interest

M.O. serves as a medical advisor for Taisho Pharmaceutical Co. and receives an advisory fee for this study. M.O. also receives lecture fees from Eli Lilly Japan K.K. and Pfizer Inc., advisory fees from Eli Lilly Japan K.K., Pfizer Inc., Bristol Myers Squibb K.K., Abbvie G.K., Sanofi K.K., Kyowa Krin Co., and RHOTO Pharmaceutical Co. and research grants from Maruho Co., Sun Pharma Japan Ltd., ADVANTEST Co., and Shiseido Co. for the projects on hair diseases but not directly related to the current work. K.A., Y.U., and T.F. are paid employees of Taisho Pharmaceutical Co., the distributor of topical minoxidil products in Japan. R.I., M.U., and M.M. served as expert dermatologists in this study and received an honorarium, and their department receives scholarship donations from Taisho Pharmaceutical Co. M.U. also received lecture fees from Eli Lilly Japan K.K., Pfizer Inc., and Abbvie G.K., and advisory fees from Pfizer Inc. M.O. is an Editorial Board member of the Journal of Dermatology and a co‐author of this article. To minimize bias, they were excluded from all editorial decision‐making related to the acceptance of this article for publication.

## Supporting information


**Figure S1.** Hair sample images for the hair wash test assessment. The number in the top left corner indicate the hair count within each red circle.

**Figure S2.** Changes in telogen hair ratio and individual trends. Individual progression of the proportion of telogen hairs after 5% minoxidil topical lotion administration for telogen effluvium (*n* = 12).

**Table S1.** Efficacy summary by subject.

## Data Availability

The data that supports the findings of this study are available in the supplementary material of this article.
