# Supplementary figures and images for: Use of 5% Topical Minoxidil Application for Telogen Effluvium: An Open‐Label Single‐Arm Clinical Trial
Source: J Dermatol. 2025 Jul 2;52(9):1351–9. doi: 10.1111/1346-8138.17844 (PMC12411807; doi:10.1111/1346-8138.17844)

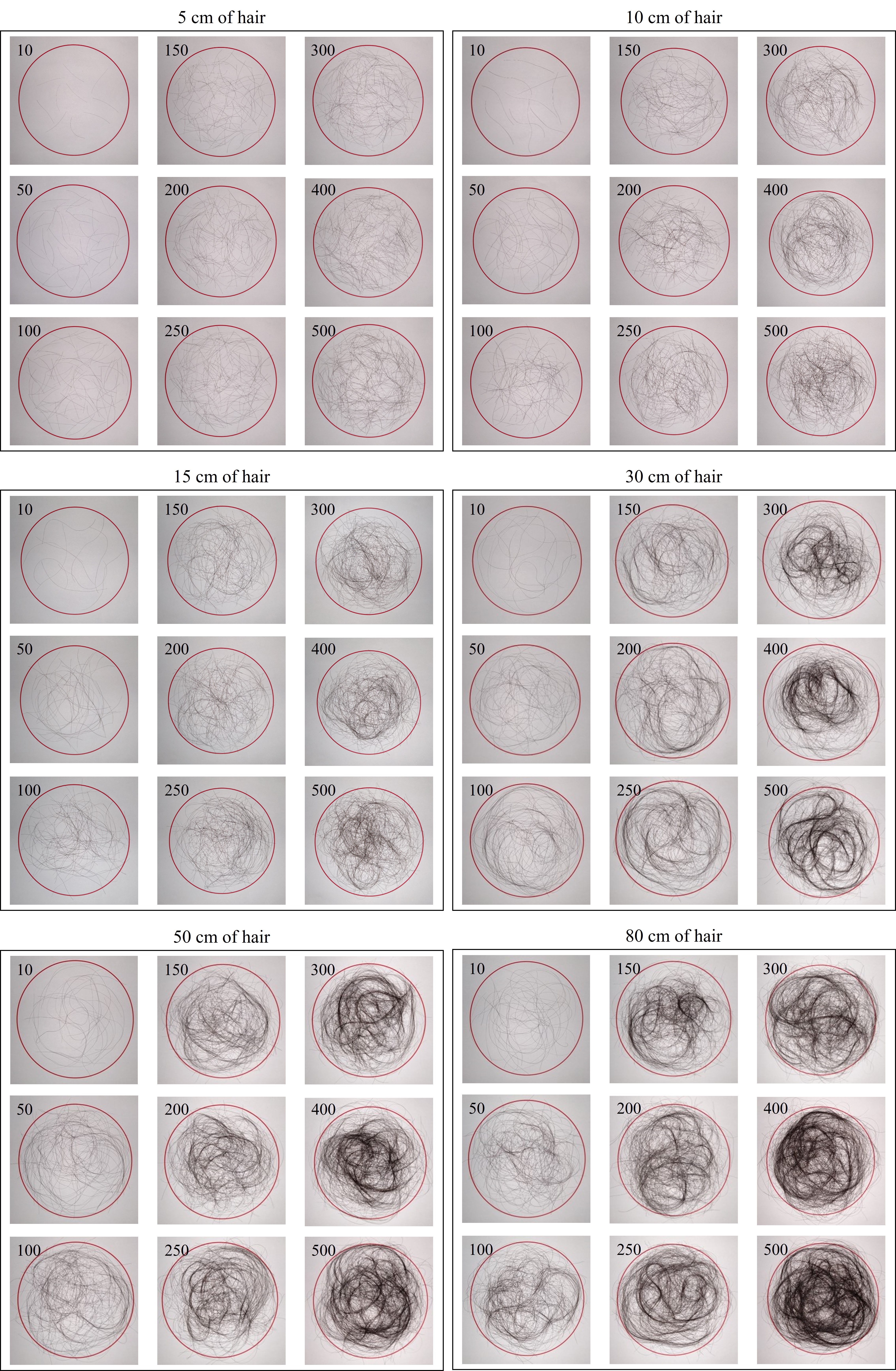

Supplement: Supplementary file 1 — Figure S1. Hair sample images for the hair wash test assessment. The number in the top left corner indicate the hair count within each red circle. [file JDE-52-1351-s002.jpg]

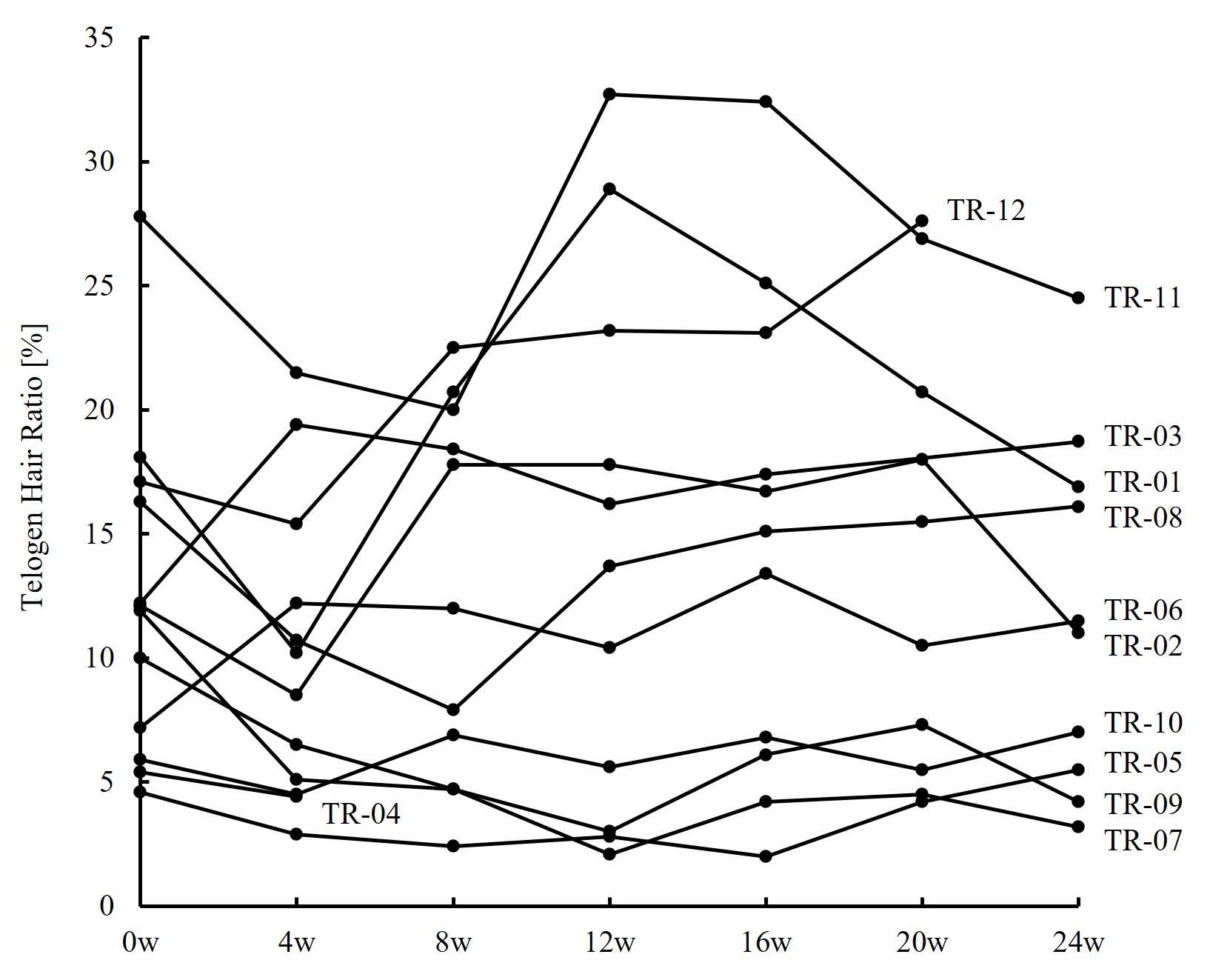

Supplement: Supplementary file 2 — Figure S2. Changes in telogen hair ratio and individual trends. Individual progression of the proportion of telogen hairs after 5% minoxidil topical lotion administration for telogen effluvium (n = 12). [file JDE-52-1351-s001.jpg]
