# Supplementary material for: Use of 5% Topical Minoxidil Application for Telogen Effluvium: An Open‐Label Single‐Arm Clinical Trial
Source: J Dermatol. 2025 Jul 2;52(9):1351–9. doi: 10.1111/1346-8138.17844 (PMC12411807; doi:10.1111/1346-8138.17844)
Supplement: Supplementary file 3 — Table S1. Efficacy summary by subject. [file JDE-52-1351-s003.docx]

**Supplementary Table S1. Efficacy Summary by Subject**

The evaluation area of each baseline was matched with the corresponding site at each evaluation time point.

| **Subject**  **ID** | **Sex** | **Age** | **Week** | **Hair Count** | | | | | | | | | | **Telogen Hair Ratio [%]** |
| --- | --- | --- | --- | --- | --- | --- | --- | --- | --- | --- | --- | --- | --- | --- |
|  |  |  |  | **Week 0 (baseline)** | | | | | **Evaluation Time Point** | | | | |  |
|  |  |  |  | **Non-vellus [/cm^2^]** | **Vellus [/cm^2^]** | **Terminal [/cm^2^]** | **Non-terminal [/cm^2^]** | **Total [/cm^2^]** | **Non-vellus [/cm^2^]** | **Vellus [/cm^2^]** | **Terminal [/cm^2^]** | **Non-terminal [/cm^2^]** | **Total [/cm^2^]** |  |
| TR-01 | Male | 48 | 0 | 107.8 | 54.1 | 79.7 | 82.2 | 161.9 | － | － | － | － | － | 18.1 |
|  |  |  | 4 | 111.1 | 59.2 | 83.3 | 86.9 | 170.3 | 161.5 | 14.4 | 120.9 | 55.0 | 175.9 | 10.2 |
|  |  |  | 8 | 113.2 | 57.6 | 84.1 | 86.7 | 170.8 | 169.8 | 16.3 | 132.1 | 54.1 | 186.1 | 20.7 |
|  |  |  | 12 | 111.8 | 58.5 | 83.3 | 87.0 | 170.3 | 158.9 | 21.2 | 119.0 | 61.1 | 180.1 | 28.9 |
|  |  |  | 16 | 111.0 | 58.3 | 83.9 | 85.4 | 169.3 | 147.8 | 25.6 | 111.0 | 62.4 | 173.4 | 25.1 |
|  |  |  | 20 | 111.9 | 56.9 | 83.2 | 85.6 | 168.8 | 143.6 | 34.2 | 106.4 | 71.3 | 177.7 | 20.7 |
|  |  |  | 24 | 112.5 | 57.3 | 83.9 | 85.9 | 169.8 | 146.1 | 34.6 | 109.1 | 71.6 | 180.7 | 16.9 |
| TR-02 | Male | 50 | 0 | 167.3 | 28.1 | 142.7 | 52.7 | 195.4 | － | － | － | － | － | 12.1 |
|  |  |  | 4 | 174.9 | 30.4 | 144.5 | 60.8 | 205.3 | 186.5 | 23.8 | 160.2 | 50.2 | 210.3 | 8.5 |
|  |  |  | 8 | 173.4 | 31.9 | 143.1 | 62.2 | 205.3 | 172.9 | 29.8 | 149.6 | 53.1 | 202.7 | 17.8 |
|  |  |  | 12 | 174.7 | 28.5 | 144.7 | 58.6 | 203.3 | 169.1 | 25.5 | 149.8 | 44.8 | 194.6 | 17.8 |
|  |  |  | 16 | 174.0 | 28.7 | 143.7 | 59.0 | 202.8 | 156.1 | 26.2 | 135.0 | 47.2 | 182.2 | 16.7 |
|  |  |  | 20 | 174.1 | 28.7 | 144.4 | 58.4 | 202.8 | 158.1 | 17.6 | 137.7 | 38.0 | 175.8 | 18.0 |
|  |  |  | 24 | 173.9 | 28.4 | 144.0 | 58.3 | 202.3 | 147.1 | 29.4 | 119.7 | 56.8 | 176.5 | 11.0 |
| TR-03 | Female | 45 | 0 | 164.9 | 69.9 | 133.9 | 100.9 | 234.7 | － | － | － | － | － | 12.2 |
|  |  |  | 4 | 174.8 | 67.8 | 135.6 | 107.0 | 242.6 | 193.2 | 47.9 | 150.9 | 90.2 | 241.1 | 19.4 |
|  |  |  | 8 | 173.6 | 68.6 | 136.7 | 105.5 | 242.2 | 183.1 | 65.4 | 156.2 | 92.3 | 248.5 | 18.4 |
|  |  |  | 12 | 174.1 | 67.5 | 135.0 | 106.6 | 241.6 | 203.0 | 43.3 | 160.2 | 86.0 | 246.3 | 16.2 |
|  |  |  | 16 | 173.8 | 67.3 | 136.7 | 104.4 | 241.1 | 192.6 | 59.0 | 156.6 | 95.0 | 251.6 | 17.4 |
|  |  |  | 24 | 174.0 | 67.2 | 136.4 | 104.8 | 241.2 | 204.6 | 45.3 | 158.3 | 91.6 | 249.9 | 18.7 |
| TR-04 | Female | 56 | 0 | 128.0 | 50.2 | 54.6 | 123.5 | 178.1 | － | － | － | － | － | 5.4 |
|  |  |  | 4 | 137.8 | 42.3 | 52.7 | 127.4 | 180.1 | 169.2 | 12.9 | 89.1 | 93.0 | 182.1 | 4.4 |
| TR-05 | Male | 36 | 0 | 165.4 | 39.9 | 149.6 | 55.6 | 205.2 | － | － | － | － | － | 4.6 |
|  |  |  | 4 | 180.4 | 28.2 | 157.9 | 50.7 | 208.7 | 190.9 | 18.8 | 180.4 | 29.3 | 209.7 | 2.9 |
|  |  |  | 8 | 179.6 | 29.6 | 158.0 | 51.2 | 209.2 | 197.0 | 16.9 | 186.5 | 27.5 | 213.9 | 2.4 |
|  |  |  | 12 | 181.5 | 28.2 | 159.8 | 49.9 | 209.7 | 200.6 | 13.1 | 184.5 | 29.2 | 213.7 | 2.8 |
|  |  |  | 16 | 181.0 | 28.2 | 159.5 | 49.7 | 209.2 | 198.5 | 12.8 | 183.6 | 27.7 | 211.3 | 2.0 |
|  |  |  | 20 | 180.9 | 28.3 | 159.3 | 49.8 | 209.1 | 204.5 | 10.8 | 186.5 | 28.8 | 215.3 | 4.2 |
|  |  |  | 24 | 180.4 | 27.3 | 157.7 | 50.0 | 207.7 | 198.1 | 10.6 | 181.9 | 26.8 | 208.7 | 5.5 |
| TR-06 | Female | 63 | 0 | 127.0 | 32.5 | 96.5 | 63.0 | 159.4 | － | － | － | － | － | 7.2 |
|  |  |  | 4 | 128.4 | 31.1 | 94.8 | 64.7 | 159.4 | 130.4 | 34.6 | 108.5 | 56.5 | 165.1 | 12.2 |
|  |  |  | 8 | 127.2 | 32.3 | 94.4 | 65.1 | 159.5 | 144.1 | 32.8 | 117.2 | 59.6 | 176.9 | 12.0 |
|  |  |  | 12 | 126.1 | 33.8 | 93.3 | 66.6 | 159.9 | 145.3 | 37.3 | 118.1 | 64.6 | 182.6 | 10.4 |
|  |  |  | 16 | 127.3 | 32.7 | 94.6 | 65.4 | 160.0 | 148.9 | 34.2 | 121.7 | 61.4 | 183.1 | 13.4 |
|  |  |  | 20 | 127.3 | 32.7 | 94.1 | 65.9 | 160.0 | 145.4 | 34.7 | 119.7 | 60.4 | 180.1 | 10.5 |
|  |  |  | 24 | 127.6 | 32.4 | 94.7 | 65.3 | 160.0 | 153.4 | 30.9 | 120.0 | 64.3 | 184.3 | 11.5 |
| TR-07 | Female | 42 | 0 | 195.9 | 37.9 | 135.8 | 97.9 | 233.8 | － | － | － | － | － | 10.0 |
|  |  |  | 4 | 203.3 | 32.0 | 132.1 | 103.2 | 235.3 | 215.2 | 21.7 | 162.5 | 74.3 | 236.8 | 6.5 |
|  |  |  | 8 | 203.2 | 32.1 | 128.3 | 107.0 | 235.3 | 218.2 | 25.7 | 162.6 | 81.3 | 243.9 | 4.7 |
|  |  |  | 12 | 203.2 | 31.6 | 128.9 | 105.9 | 234.8 | 217.1 | 26.2 | 148.7 | 94.7 | 243.3 | 2.1 |
|  |  |  | 16 | 203.8 | 32.0 | 131.5 | 104.2 | 235.7 | 225.8 | 18.3 | 155.6 | 88.5 | 244.1 | 4.2 |
|  |  |  | 20 | 202.4 | 32.4 | 130.2 | 104.6 | 234.8 | 233.3 | 9.9 | 179.9 | 63.3 | 243.2 | 4.5 |
|  |  |  | 24 | 204.1 | 31.2 | 132.7 | 102.6 | 235.3 | 222.3 | 26.0 | 155.6 | 92.7 | 248.3 | 3.2 |
| TR-08 | Female | 55 | 0 | 191.4 | 48.7 | 126.5 | 113.7 | 240.2 | － | － | － | － | － | 16.3 |
|  |  |  | 4 | 189.6 | 53.5 | 123.1 | 120.1 | 243.1 | 186.1 | 58.5 | 123.6 | 121.1 | 244.6 | 10.7 |
|  |  |  | 8 | 190.4 | 55.7 | 124.1 | 122.0 | 246.1 | 184.3 | 76.5 | 121.5 | 139.2 | 260.8 | 7.9 |
|  |  |  | 12 | 189.5 | 57.1 | 125.3 | 121.2 | 246.6 | 205.3 | 66.2 | 130.9 | 140.6 | 271.5 | 13.7 |
|  |  |  | 16 | 188.7 | 58.3 | 123.3 | 123.8 | 247.1 | 199.0 | 62.4 | 131.5 | 129.9 | 261.4 | 15.1 |
|  |  |  | 20 | 189.4 | 56.7 | 122.8 | 123.3 | 246.1 | 200.3 | 60.4 | 131.6 | 129.0 | 260.7 | 15.5 |
|  |  |  | 24 | 191.2 | 55.4 | 124.3 | 122.3 | 246.6 | 200.7 | 61.4 | 140.3 | 121.8 | 262.1 | 16.1 |
| TR-09 | Female | 57 | 0 | 194.9 | 9.8 | 169.3 | 35.4 | 204.7 | － | － | － | － | － | 11.9 |
|  |  |  | 4 | 196.7 | 12.6 | 169.5 | 39.7 | 209.2 | 186.2 | 17.3 | 165.8 | 37.7 | 203.5 | 5.1 |
|  |  |  | 8 | 196.9 | 12.7 | 169.4 | 40.2 | 209.6 | 190.0 | 14.3 | 164.1 | 40.2 | 204.3 | 4.7 |
|  |  |  | 12 | 196.7 | 13.4 | 168.8 | 41.3 | 210.1 | 199.3 | 17.0 | 170.9 | 45.4 | 216.3 | 3.0 |
|  |  |  | 16 | 196.2 | 13.5 | 169.3 | 40.4 | 209.6 | 204.5 | 14.5 | 174.9 | 44.0 | 218.9 | 6.1 |
|  |  |  | 20 | 198.1 | 12.5 | 171.5 | 39.1 | 210.6 | 201.8 | 19.8 | 175.2 | 46.4 | 221.6 | 7.3 |
|  |  |  | 24 | 197.5 | 12.7 | 169.5 | 40.7 | 210.1 | 205.9 | 7.9 | 182.7 | 31.2 | 213.8 | 4.2 |
| TR-10 | Female | 42 | 0 | 220.0 | 19.2 | 190.0 | 49.2 | 239.2 | － | － | － | － | － | 5.9 |
|  |  |  | 4 | 220.4 | 23.8 | 194.5 | 49.7 | 244.2 | 218.8 | 23.3 | 196.6 | 45.5 | 242.1 | 4.5 |
|  |  |  | 8 | 220.1 | 23.5 | 195.1 | 48.5 | 243.6 | 221.7 | 19.3 | 194.1 | 46.9 | 241.0 | 6.9 |
|  |  |  | 12 | 219.8 | 24.4 | 193.9 | 50.3 | 244.2 | 224.9 | 14.2 | 195.4 | 43.7 | 239.1 | 5.6 |
|  |  |  | 16 | 218.9 | 24.8 | 195.1 | 48.5 | 243.7 | 212.9 | 35.6 | 191.4 | 57.1 | 248.5 | 6.8 |
|  |  |  | 20 | 221.0 | 23.1 | 196.9 | 47.2 | 244.1 | 227.8 | 19.9 | 202.6 | 45.1 | 247.8 | 5.5 |
|  |  |  | 24 | 219.6 | 23.5 | 196.1 | 46.9 | 243.1 | 213.4 | 27.1 | 193.0 | 47.5 | 240.5 | 7.0 |
| TR-11 | Female | 33 | 0 | 108.8 | 50.2 | 86.1 | 72.8 | 159.0 | － | － | － | － | － | 27.8 |
|  |  |  | 4 | 105.1 | 55.3 | 86.7 | 73.7 | 160.4 | 103.6 | 50.3 | 84.7 | 69.3 | 154.0 | 21.5 |
|  |  |  | 8 | 104.6 | 56.8 | 86.9 | 74.5 | 161.4 | 105.1 | 61.7 | 82.9 | 83.9 | 166.8 | 20.0 |
|  |  |  | 12 | 104.6 | 56.8 | 87.4 | 74.0 | 161.4 | 100.7 | 70.1 | 83.4 | 87.4 | 170.8 | 32.7 |
|  |  |  | 16 | 105.0 | 57.0 | 86.7 | 75.3 | 162.0 | 108.5 | 61.4 | 79.7 | 90.1 | 169.9 | 32.4 |
|  |  |  | 20 | 105.2 | 55.3 | 86.9 | 73.6 | 160.5 | 99.8 | 56.8 | 79.0 | 77.5 | 156.5 | 26.9 |
|  |  |  | 24 | 104.8 | 56.1 | 87.1 | 73.8 | 160.9 | 97.9 | 59.5 | 79.2 | 78.2 | 157.5 | 24.5 |
| TR-12 | Female | 45 | 0 | 217.0 | 66.9 | 133.9 | 150.1 | 284.0 | － | － | － | － | － | 17.1 |
|  |  |  | 4 | 202.9 | 87.5 | 132.3 | 158.1 | 290.4 | 179.5 | 109.9 | 114.4 | 175.0 | 289.4 | 15.4 |
|  |  |  | 8 | 202.1 | 87.9 | 132.8 | 157.1 | 289.9 | 195.9 | 111.1 | 116.8 | 190.2 | 307.0 | 22.5 |
|  |  |  | 12 | 201.5 | 87.8 | 131.5 | 157.9 | 289.3 | 191.4 | 117.8 | 114.2 | 194.9 | 309.1 | 23.2 |
|  |  |  | 16 | 200.5 | 89.8 | 131.0 | 159.4 | 290.4 | 204.6 | 101.5 | 121.3 | 184.8 | 306.1 | 23.1 |
|  |  |  | 20 | 202.3 | 87.1 | 131.2 | 158.2 | 289.4 | 194.3 | 102.2 | 121.2 | 175.3 | 296.4 | 27.6 |
